# Laparoscopic simulator performance and learning curves under different optical angles

**DOI:** 10.1186/s12909-023-04555-z

**Published:** 2023-08-29

**Authors:** Bas Kengen, Harry van Goor, Jan-Maarten Luursema

**Affiliations:** Department of Surgery, Radboud University Medical Center, PO Box 9101 (960), Nijmegen, 6500 HB The Netherlands

**Keywords:** Laparoscopy, Training, Laparoscopic simulator, Optical angle

## Abstract

**Background:**

Deviated optical angles create visuospatial and psychomotor challenges during laparoscopic procedures, resulting in delayed operative time and possibly adverse events. If it is possible to train the skills needed to work under these deviated optical angles, this could benefit procedure time and patient safety. This study investigates the influence of the optical angle on development of basic laparoscopic surgical skills.

**Methods:**

A total of 58 medical students performed a four-session laparoscopic training course on a Virtual Reality Simulator. During each session, they performed an identical task under optical angles of 0°, 45° and − 45°. Performance parameters of task duration and damage were compared between the optical angles to investigate the effect of optical angle on performance development. The 4th session performance was compared to the 2nd session performance for each angle to determine improvement.

**Results:**

Participants performed the task significantly faster under the 0° optical angle compared to the plus and minus 45° optical angles during the last three sessions (*z* between − 2.95 and − 2.09, *p* < .05). Participants improved significantly and similarly for task duration during the training course under all optical angles. At the end of the training course however significant performance differences between the zero and plus/minus 45 optical angles remained. Performance for damage did not improve and was not affected by optical angle throughout the course.

**Conclusion:**

Dedicated virtual reality training improves laparoscopic basic skills performance under deviated optical angles as it leads to shorter task duration, however a lasting performance impairment compared to the 0° optical angle remained. Training for performing under deviating optical angles can potentially shorter the learning curve in the operating room.

## Introduction

Laparoscopic surgery is relatively hard to learn, which is demonstrated by studies that show longer learning curves for laparoscopic procedures compared to open surgery [1, 2]. Visuospatial and psychomotor challenges inherent in working with indirect vision and over a fulcrum are important contributors to this extended learning curve [3–5], especially when working under deviated optical angles [6–8]. This latter challenge has not been structurally addressed in laparoscopic simulator training courses.

**Fig. 1:**
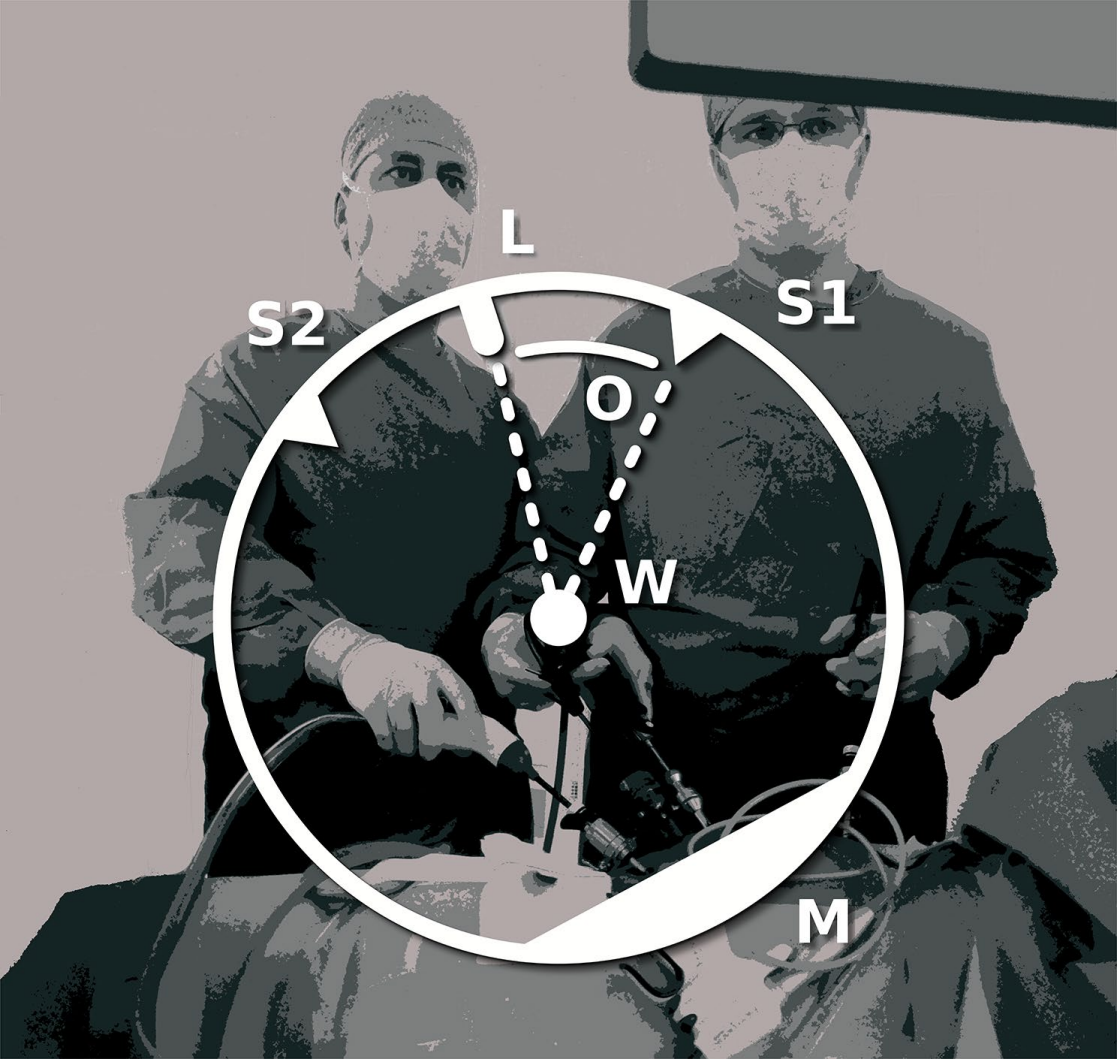
A laparoscopic procedure in the operating room with a corresponding schematic helicopter view to show the variables relevant to the challenges of laparoscopic indirect vision and optical angle (the angle between the laparoscope and the line vision of the surgeon towards the working field). O = optical angle, S1 = surgeon 1, S2 = surgeon 2, L = Laparoscope, W = working field, M = monitor

The optical angle is defined as the angle between the line of action (the horizontal projection of the line connecting the trocar for the laparoscope to the anatomical target) and the line of vision (the horizontal projection of the line connecting the central axis of the surgeon with the anatomical target) (Fig. 1) [9]. For reasons of anatomy, pathology, team placement, and procedural techniques such as switching the camera to a different trocar, it is not always possible to maintain the optimal optical angle of 0°. To reduce the risks and effort of placing an extra trocar, a deviated angle is sometimes deemed acceptable. Previous research demonstrated longer task duration for deviated optical angles during the performance of simulated laparoscopic tasks [9–11]. In these studies, with both novices and experts, all participants showed a decrease in performance under deviated optical angles. However, experienced participants showed better adaption to deviated optical angles, as their performance was both relatively and absolutely less affected by a deviated optical angle compared to novice participants. To our knowledge no previous studies have investigated the learning curves for (simulated) laparoscopic performance under deviated optical angles.

During actual laparoscopic surgery, performance on both time and damage for inexperienced surgeons is likely to be impacted more negatively by non-zero optical angles compared to more experienced surgeons. Yet, many current simulation curricula do not include training skills under non-zero optical angles. This is reflected in the design of videobox trainers and virtual reality simulators, most of which do not have features to facilitate such training. The question arises how we could better prepare laparoscopic novices for their first encounter with deviated optical angles in the operating room, i.e. could this be realized with simulation training and how would these laparoscopic skills develop. With effective simulation training the learning curve for non-zero laparoscopic optical angles in the operating room could possibly be shortened, improving patient safety, as most complications occur during the first 30–50 laparoscopic procedures of a surgeon [12–15].

In this study we compare the development of laparoscopic skills under a standard zero optical angle to optical angles of plus and minus 45 degrees. Primary endpoints were damage and task duration. Based on previous research, we hypothesize better performance for working under an optical angle of 0° in comparison to deviated optical angles. We also expect working under deviated optical angles can be trained and that differences in performance under different optical angles will attenuate with training.

## Materials and methods

### Subjects and course design

Data for this study was collected over a period of 4 months at the Surgical department of the Radboud University Medical Center, the Netherlands. Here, every month a new cohort of first year master students of Medicine starts the surgical internship. At the time of data collection, students could opt to take a voluntary, four-session basic skills laparoscopic simulator training course as part of a 3-week preparatory course for this internship. Every month, between 19 and 29 students enrolled in this training course. Power calculations based on effect sizes reported by Haveran and colleagues for differences in performance for task duration between a 0° optical angle and 60° optical angle revealed that 34 participants were needed to achieve a power of 0.8 [9]. Four cohorts were included with a total of 58 participants, to ensure sufficient participants who completed all four sessions, as we saw a high drop-out percentage during previous studies [16]. Written informed consent was obtained from all participants and it was made clear to the participants that their data would be analyzed anonymously for scientific purposes only, within our research group. We also made it clear that not consenting to our using their training data would in no way impact their training course experience or any assessments later. All methods were carried out in accordance with relevant guidelines and regulations. No formal ethics review was sought, as this was not required under Dutch law for this type of research at the time of data collection [17].

To take part in the course, students could register themselves online in groups of three for a preferred time and date. Only one session per day was allowed to maintain a distributed practice schedule, as it is known that this leads to better retention of psychomotor skills [18, 19]. Training sessions were always finished within a 60-minute time-frame.

### Training session

During the first session, each subject completed a digital demographics and laparoscopic experience questionnaire. Also, a short instruction was given during the first session to introduce students to the skillslab, simulators, and the various training tasks. Supervision was given throughout the whole first session. On request, supervision was available for the last three sessions. This opportunity was not used.

At the LapSim, students started every session with a warming-up exercise under a 0°, 45° and − 45° optical angle (Camera Navigation). During this exercise students are in control of the laparoscope and have to focus the camera centrally on multiple digital gallstones spread throughout a virtual abdominal cavity. After this they performed the task ‘Grasping’ three times under different optical angles: 0°, 45° and − 45°. The task was always performed in this order of optical angles. We chose this order because a pilot study showed us that it was too hard for most students to start learning the tasks with an optical angle of 45° or -45°, and they could not finish the task within an acceptable time window (data not shown). We chose the exercise ‘Grasping’ because it is moderately complex, so we expected the students to cause damage, but still show a (nearly) complete learning curve after completing the course. During this task participants use a virtual laparoscopic grasper with actual handles alternating between the left and right hand, to retract tubular structures and place these into a retrieval bag in a simulated abdominal cavity. More information about the performed tasks can be found on the website of Surgical Science [20].

In the skillslab participants also had access to an in-house developed videobox trainer. On this trainer students could use standard laparoscopic instruments to perform simple psychomotor tasks. Training activity on this videobox trainer was not monitored.

### Apparatus/materials

**Fig. 2:**
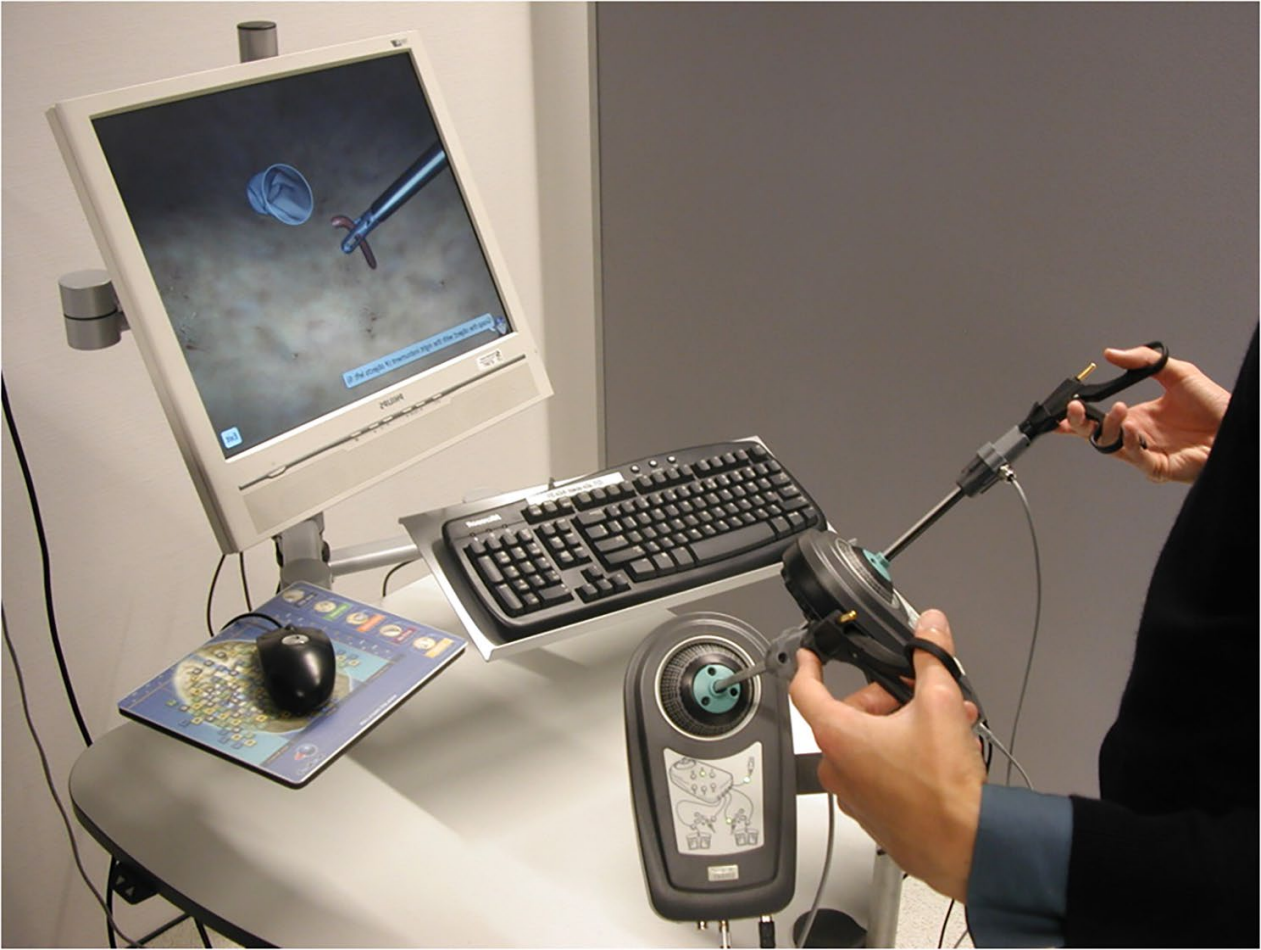
Picture of the training station, consisting of Surgical Science’s LapSim.

The training station consisted of a desktop running windows, a laparoscopic interface consisting of the Simball hardware (G-coder Systems, Västra Frölunda, Sweden) and Surgical Science’s LapSim v3.0 training software (Surgical Science, Göteborg, Sweden)(Fig. 2). This software contained multiple exercises, but the students could only use the exercise ‘Grasping’ in the three optical angles. Questionnaires were created and completed with LimeSurvey v1.92+, a web-based application to create surveys and collect responses. The questionnaires were completed on-site, on an Asus Laptop running Windows 7. The IBM SPSS Statistics v.23 package was used for data analysis.

### Data preparation

During the performance of laparoscopic tasks, the LapSim simulator automatically records performance parameters such as task duration, instrument path length, angular path, tissue damage, and maximum damage. Since our primary endpoints were task duration and damage, the parameters of task duration, tissue damage, and maximum damage were included in the data analysis. Task duration was recorded in milliseconds. Tissue damage reports the number of times damage was caused by injuring the virtual abdominal cavity, while maximum damage represents the depth of damage created in millimeters for the most severe collision of instrument and virtual tissue. In the current available literature, there is still uncertainty regarding the interpretation of increased path length or angular path. On one hand, they could be negatively associated with less efficient movement. On the other hand, they could be positively associated with additional safety measures, such as avoiding critical anatomy and ensuring visibility of laparoscopic instruments. Furthermore, we expected these parameters to correlate with task duration, suggesting potential redundancy. Consequently, these parameters were not included in the data analysis. Extreme outliers (data points that exceeded the 90th percentile) were removed from the dataset. This led to a data loss of 5,7%.

To compare improvement in performance during the course between the different optical angles, we created ‘improvement variables’ by subtracting performance at the 4th session from performance at the 2nd session. We chose the second rather than the first session to create these new variables, reasoning that during the first session performance is slowed by adapting to a novel training environment (rather than reflecting laparoscopic performance per se).

### Data Analysis

Shapiro-Wilk tests showed that not all parameters followed a normal distribution because of a floor effect for damage. Wilcoxon signed-rank tests were therefore used to analyze the data.

To assess the effect of optical angle on performance, we used pairwise comparisons for performance under the 0°, 45° and − 45° optical angles for task duration, tissue damage, and maximum damage, respectively. This was done for every session separately. We also compared performance at the 4th session to performance at the 2nd session for every optical angle to see if participants improved during the course. To assess if this improvement in performance differed between the optical angles, we additionally compared improvement variables between the different optical angles. A level of *p* < .05 was considered statistically significant for all tests.

## Results

### Participants

All 58 students who participated to the training course volunteered to participate in the research. The 43 students who completed the (voluntary) course were included for data analysis (34 female and 9 male, average age of 24 years ranging from 21 to 30 years). None of the participants reported any previous laparoscopic experience.

### Performance

**Fig. 3:**
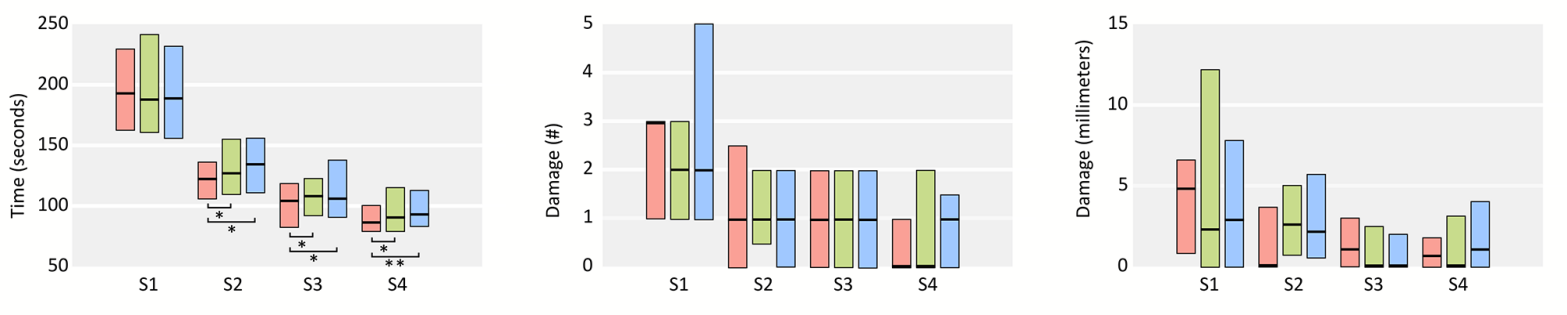
Boxplots showing median and interquartile range for performance for each session, for the 0° optical angle (red), 45° optical angle (green) and − 45° optical angle (blue). From left to right: task duration (in seconds), frequency of tissue damage, and maximum damage in millimeters. * = p < .05, ** = p < .01

A summary of all performance parameters is shown in Fig. 3. Students performed faster at the 0° optical angle compared to either the 45° or -45° optical angle for all but the first sessions (*z* between − 2.95 and − 2.09, *p* < .05). We found no significant performance difference for task duration between 45° and − 45° (Table 1). Comparison between the 4th and 2nd training session demonstrated that individuals significantly improved (*z* between − 0.40 and − 0.17, *p* < .05) in task duration under all three optical angles (Table 1).

**Table 1:** Median task duration in seconds per session in paired comparisons between the three optical angles. The last row demonstrates the median difference in task duration in seconds between the 2nd and 4th session, reflecting improvement in performance during the course. Wilcoxon Signed Ranks tests were performed to compare task duration between the different optical angles, z values and p values are shown. Also, Wilcoxon Signed Ranks tests were performed to compare improvement in task duration during the course between the optical angles, shown in the last row. * = Significant

Session	0°	45°	*z* value	*p* value	0°	-45°	*z* value	*p* value	45°	-45°	*p* value	*z* value
1	193.4	188.4	-0.16	0.88	193.4	189.2	-0.75	0.45	188.4	189.2	0.66	-0.45
2	122.0	127.1	-2.09	0.04*	122.0	134.0	-2.36	0.02*	127.1	134.0	0.71	-0.37
3	103.6	107.7	-2.17	0.03*	103.6	105.5	-2.54	0.01*	107.7	105.5	0.60	-0.53
4	85.2	90.2	-2.38	0.02*	85.2	92.3	-2.95	0.00*	90.2	92.3	0.67	-0.42
Difference Session 2–4	-31.5	-38.9	-0.40	0.69	-31.5	-28.8	-0.17	0.87	-38.9	-28.8	0.79	-0.27

Both tissue damage and maximum damage did not differ significantly in performance between the three optical angles any of the sessions (Fig. 3). The participants did not perform significantly better for both tissue damage or maximum damage during the 4th session compared to the 2nd session under any optical angle (Fig. 3). Also, no differences in improvement between the 2nd and 4th session between the different optical angles were found.

## Discussion

During a four-session basic skills laparoscopic training course to support medical students prepare for their surgical rotations, better performance was demonstrated for duration but not for damage under a 0° optical angle compared to 45° and − 45° optical angles. For duration, performance improved for all three angles. However, we found no attenuation for the performance differences between the angles over the sessions. No significant improvements for the damage variables were found.

The lack of attenuation of performance differences over time surprised us, as we initially expected that with sufficient training, performance under non-zero optical angles would eventually match that of the zero degree angle. While more experienced surgeons demonstrated relatively better performance under deviated optical angles compared to inexperienced surgeons ([9–11]), they still fell short when compared to the performance under the zero degree angle. It remains uncertain whether these more experienced surgeons, like our study participants, simply did not reach the end of the learning curve, and if differences in performance only emerge at a later stage of the curve. To gain further insights into the development of spatial skills associated with laparoscopic surgery, it will be necessary to study longer segments of the learning curve. Understanding the extent to which spatial skills can be trained is crucial for surgical education and the design of laparoscopic procedures. If spatial skills can be adequately trained, it should be incorporated as an explicit component of the curriculum. However, if not, additional efforts should be made to minimize the use of optical angles that deviate from zero during laparoscopic surgery.

Our lack of findings with regards to the effect of training and optical angle on damage parameters reflects a floor effect. This may have been caused by students taking extra time to complete the task to avoid creating extra damage during these tasks [18]. For future research, tasks and task settings that make it harder to avoid creating damage would be necessary to gain more insight in the learning curve of these clinically relevant parameters.

Our results partly confirm previous single-session studies that compared optical angle with surgical simulator performance. These studies found progressive deterioration in performance on time and accuracy for simulated laparoscopic tasks under increasing optical angles [9–11]. To our knowledge no previous studies have investigated the learning curve of laparoscopic skills under non-zero optical angles. However, in a two-session dentistry study the development of psychomotor skills in a simulated environment was evaluated with indirect vision under a 180° mirrored image [21]. They found significant performance improvements, demonstrating the possibility to train the skills needed to work under a deviated optical angle. They did not compare the improvement to a 0° optical angle. Also, the procedures in dentistry lack a fulcrum effect which is present in laparoscopic surgery, which impedes direct comparison.

Previous studies in laparoscopic surgery demonstrated that even for experienced surgeons performance decreases under non-zero optical angles, however experienced surgeons showed better adaptation compared to novices [9–11]. This suggests a learning effect for surgeons for working under non-zero optical angles, although with a persisting performance penalty, which is in accordance with our findings. To optimize training course length for clinically relevant optical angles and to learn more about spatial skills development in general, we need more medium- and long-term studies into performance development under these angles. Given the relevance of visuospatial ability for learning and performing highly spatial skills such as those needed for laparoscopy [4, 22, 23], we would advise visuospatial ability testing to become a standard feature of such research.

### Strengths and limitations

Using performance of simulated laparoscopic tasks on a well validated virtual reality simulator allowed for objective and standardized measurements. Implementing multiple training sessions in our study protocol enabled us to monitor the learning curve under different optical angles. Study participants were inexperienced, which provided us an unbiased visualization of this learning curve. These factors made it possible to compare laparoscopic basic skills development between the 0° and (+/-) 45° optical angles.

During our study, participants always performed the task first under a 0° optical angle before performing it under deviated angles. Since learning is expected to occur during the exercise, the performance difference between the 0° and deviated optical angles may have been smaller compared to a truly randomized design. This effect is expected to be at its largest during the first session, because students still had to learn how to execute the task and how to work with the simulator. Despite this disadvantage, the participants still performed significantly better for time under a 0° optical angle during the last three sessions, demonstrating the relevance of the effect of optical angle.

During this basic skills laparoscopic training course, students were free to train on an in-house developed laparoscopic videobox trainer. This trainer was not part of the study and activity was not monitored, however could have affected the performance on the LapSim, as the tasks for this simulator are designed to train similar skills. Some transfer of skills may have occurred however which could have led to improved performance on the LapSim tasks. This effect is expected to be equal for the 0°, 45° and − 45° optical angle and thus unlikely to affect our conclusions.

We specifically concentrated on the parameters related to task duration and damage to assess their impact on laparoscopic performance. While there may be additional parameters that could also influence performance, the power calculation and group size limitations prevented us from conducting subgroup analysis for those factors.

### Impact

Our results confirm that performing laparoscopic tasks under non-zero degree optical angles is more challenging compared to a zero degree optical angle ([9–11]). New in comparison to previous studies is that while it is possible to train and improve the necessary skills for such tasks, a performance gap remains between zero- and non-zero angled laparoscopic simulator performance. Currently, training curricula often focus on a zero degree optical angle, while neglecting non-zero degree angles. While questions remain regarding skill transfer and the learning curve, we recommend the implementation of laparoscopic skills training for non-zero optical angles in basic skills simulation training courses. This will better prepare young surgeons for the inevitable encounters with these angles during real surgeries. Training laparoscopic performance under non-zero optical angles in a safe environment could reduce the learning curve, and improve performance in the operating room.

### Future research

To be able to fully recommend training for relevant non-zero optical angles we need to answer a number of additional questions. One has to do with transfer of optical angle skills; if you train one task under, say, a 45° optical angle, does this shorten your learning curve for a novel task under the same 45° optical angle? Another has to with transfer of training under one optical angle to other optical angles; if you train under 45°, do you shorten the learning curve for optical angles of 90°, or 50°, or 46°, or -45°? In a separate study we found evidence for ‘zones of performance’ (optical angles with a similar performance penalty) that may translate to ‘zones of training’ (unpublished data). We would also like to study longer segments of the learning curve for a larger number of optical angles to optimize training efforts for these spatial skills, and to learn more about the extent to which these skills can be trained to match performance under a reference optical angle of 0°.

The spatial complexity of laparoscopy is not just dependent on optical angle, but also on the angle of the laparoscope’s lens system (compared to the laparoscope’s central axis). Interactions between optical angle and lens angle can further complicate laparoscopic performance. A variable of interest for all the studies proposed above is visuospatial ability, which may greatly impact the speed and end level of learning and performance for these spatial skills. We also recommend where possible to go beyond duration as primary outcome measure and study proxies for complications and damage, which are a more direct measure of skill and ultimately are likely to be more relevant to patient outcomes. This should give a better handle at both the training effort needed for learning to perform under non-zero optical angles, and for the performance penalties associated with different optical angles in a clinical setting.

## Conclusion

Performing a laparoscopic task under a deviated optical angle of (+/-) 45° induces a significant and lasting increase in task duration compared to a 0° optical angle in early basic skills training. However, novices are able to improve performance under deviated angles, therefore implementing training under deviated optical angles into basic training courses could help prepare young surgeons for real surgery and potentially shorten learning curves in the operating room.

## Data Availability

The datasets used and analyzed during the current study are available from the corresponding author on reasonable request.
